# Transcriptomic Analysis of Orange Fruit Treated with Pomegranate Peel Extract (PGE)

**DOI:** 10.3390/plants8040101

**Published:** 2019-04-17

**Authors:** Imen Belgacem, Sonia Pangallo, Ahmed Abdelfattah, Flora V. Romeo, Santa O. Cacciola, Maria G. Li Destri Nicosia, Gabriele Ballistreri, Leonardo Schena

**Affiliations:** 1 Dipartimento di Agraria, Università Mediterranea di Reggio Calabria, Località Feo di Vito, 89122 Reggio Calabria, Italy; imen.belgacem@unirc.it (I.B.); sonia.pangallo@unirc.it (S.P.); ahmed.abdelfattah@su.se (A.A.); giulia.lidestri@unirc.it (M.G.L.D.N.); 2 Department of Ecology, Environment and Plant Sciences, Stockholm University, 106 91 Stockholm, Sweden; 3 Consiglio per la ricerca in agricoltura e l’analisi dell’economia agraria (CREA) – Centro di Ricerca Olivicoltura, Frutticoltura e Agrumicoltura, Corso Savoia 190, 95024 Acireale, Italy; floravaleria.romeo@crea.gov.it (F.V.R.); gabriele.ballistreri@crea.gov.it (G.B.); 4 Dipartimento di Agricoltura, Alimentazione e Ambiente, Università degli Studi di Catania, Via S. Sofia 100, 95123 Catania, Italy; sacaccio@unict.it

**Keywords:** orange, pomegranate peel extract, PGE, RNA-seq, transcriptomics, plant defense

## Abstract

A Pomegranate Peel Extract (PGE) has been proposed as a natural antifungal substance with a wide range of activity against plant diseases. Previous studies showed that the extract has a direct antimicrobial activity and can elicit resistance responses in plant host tissues. In the present study, the transcriptomic response of orange fruit toward PGE treatments was evaluated. RNA-seq analyses, conducted on wounded fruits 0, 6, and 24 h after PGE applications, showed a significantly different transcriptome in treated oranges as compared to control samples. The majority (273) of the deferentially expressed genes (DEGs) were highly up-regulated compared to only 8 genes that were down-regulated. Gene Ontology (GO) and Kyoto encyclopedia of genes and genomes (KEGG) pathway enrichment analysis showed the involvement of 1233 gene ontology (GO) terms and 35 KEGG metabolic pathways. Among these, important defense pathways were induced and antibiotic biosynthesis was the most enriched one. These findings may explain the underlying preventive and curative activity of PGE against plant diseases.

## 1. Introduction

The peel of pomegranate, accounting for approximately 50% of the total fruit weight, is a rich source of phenolic components, including phenolic acids and flavonoids such as anthocyanins and hydrolyzable tannins. The latter compounds are mainly represented by punicalagins, ellagic acid and its derivatives [1,2]. Ellagitannins are the most important and abundant phenolic compounds in pomegranate peel and are responsible for strong antioxidative and antimicrobial activities [3,4]. Therefore, pomegranate peel extracts have recently received great attention as valuable natural compounds for a number of applications. For instance, they were proposed as effective alternative means to inhibit the germination and growth of several mammalian pathogenic bacteria including *Listeria monocytogenes, L. innocua, Staphylococcus aureus, Escherichia coli, Yersinia enterocolitica*, *Pseudomonas aeruginosa,* and *Salmonella* spp. [5,6]. 

Of particular relevance are their potential agricultural applications. An alcoholic extract from pomegranate peel, named PGE, proved high efficacy in controlling several plant diseases when applied both before and after harvest. A high level of protection was achieved against *Botrytis cinerea* on table grapes and sweet cherries, *Monilinia* spp. on sweet cherries, *Penicillium digitatum* and *Penicillium italicum* on citrus species, *Penicillium expansum* on apples and *Colletotrichum* spp. on olives [7,8,9]. In addition to its wide spectrum of activity, several other important features were reported on PGE, such as the high level of efficacy, in both preventive and curative applications, a complex mechanism of action, which includes direct fungicidal and bactericidal activities, and the capability of inducing resistance in the host tissues [9,10]. The induction of resistance in host tissues treated with PGE was indirectly demonstrated on citrus and olive fruit inoculated with *P. digitatum* and *P. italicum* [9], and *Colletotrichum acutatum* [10]. In fact, rots were also significantly reduced when no direct contact was made between the PGE and the pathogens. Furthermore, the application of PGE on grapefruits caused a significant increase of reactive oxygen species (ROS) which reached a peak after 24 h post treatment [9]. Analyses revealed the activation of several genes involved in plant defense responses such as CHI, CHS, MAPK, MAPKK, and PAL. These PGE features seem to be a direct consequence of its rich content in phenols [7]. In fact, phenolic components are potent antimicrobial agents that exert a direct effect on fungal pathogens and can also induce resistance in the plants [11]. For instance, quercetin, a common polyphenol in plant tissues induced resistance in plants and fruits by acting on the transcription level of defense genes [12,13]. The knowledge of genes and pathways involved in the induced resistance is essential to understand the mechanisms of action of PGE and may have important practical implications facilitating the development of appropriate formulation and methods of application to better control postharvest diseases [14].

Therefore, the aim of the present study was to investigate the impact of PGE on the transcriptome of treated orange fruits in order to investigate the molecular basis of the induced resistance after PGE applications.

## 2. Materials and Methods 

### 2.1. Experimental Design and Sampling 

A stock solution of PGE containing 120 g/L of dry matter and 1% citric acid used as antioxidant was obtained according to Romeo et al. (2015). The solution was stored at 5 °C and diluted just before use. 

Freshly harvested oranges (*Citrus sinensis* cv. Valencia) from organic agriculture were wounded with a sterile needle around the pedicel to produce three equidistant wounds (2 mm deep and wide) and were treated with 20 μL of PGE (12 g/L), 1% citric acid or sterile water (control). Citric acid was included in the trials since it is commonly used to stabilize PGE [7]. Samples were taken at three time intervals after treatments: 1) soon after treatment “1 h”, 2) 6 h after the treatment “6 h” 3) and 24 h after the treatment “24 h”. At each time point, albedo and flavedo were excised around the wounding sites using an 8 mm diameter cork-borer. For each treatment, three replicates, each consisting of 9 wounds from three different oranges were collected (*n* = 27). Samples were immediately frozen in liquid nitrogen, ground using a mortar and pestle, and stored at −20 °C. Total RNA was extracted from 30 mg of ground fruit tissue using the SV Total RNA Isolation System kit (Promega). The extracted RNA was purified using the DNA-free kit (Invitrogen). Each RNA sample was adjusted to have a total volume of 50 µl of total RNA. Library construction and sequencing were conducted at Macrogen Inc. (Seoul, Korea) using an Illumina Hi-Seq 2500 System to obtain 100 bp paired-end reads. Reads were deposited in the Sequence Read Archive with the accession number (PRJNA428949).

### 2.2. Data Analysis

The quality of the obtained raw reads was evaluated using the FastQC tool, version 0.11.3 (https://www.bioinformatics.babraham.ac.uk/projects/fastqc/) and was trimmed with Trimmomatic V 0.36 [15] using a 4-base wide sliding window trimming approach with an average quality of 15 and minimum read length of 36. Reads were mapped to the genome draft of sweet orange (*C. sinensis*) version 2 [16] using TopHat 2.1.1 [17]. The mapped reads were assembled into transcripts using the default setting of Cufflinks except that the library normalization method was set to geometric which employs the DESeq normalization method [18,19]. Significant changes in transcript expression were determined using Cuffdiff as implemented in Cufflinks with the default 0.05 *q*-value cut-off. Gene expression values (FPKM) were used to conduct Principal Coordinates Analysis (PCoA) and to construct heatmaps, using Qlucore v3.3 (Qlucore, Lund, Sweden) bioinformatic software. A list of significantly differentiated genes (*q*-value ≤ 0.049 corresponding to a *p*-value of 0.02187 R2 ≥ 0.2728) was selected and the corresponding sequences were extrapolated from the *Citrus sinensis* genome reference. These genes were mapped and annotated, and their Gene Ontology (GO) terms (Level 2), and pathways were analyzed using Blast2GO version 2.6.6 using default parameters [20].

## 3. Results

After quality filtering and adaptor trimming, the High-Throughput Sequencing resulted in a total of 767,487,068 sequences for read 1 and read 2 combined, and an average of 14,212,723 paired-end reads per sample (Table 1). Reads mapping on the genome draft of sweet orange (*C. sinensis*) resulted in the identification of 30,142 mapped genes.

While 30,142 genes were included in the analysis, 585 genes remained after filtering the variance according to the Qlucore software’s recommendation. Among those, 281 genes were differentially expressed (DEG) and significantly differentiated fruit treated with PGE from those treated with water and citric acid, regardless of the sampling time point.

**Figure 1:**
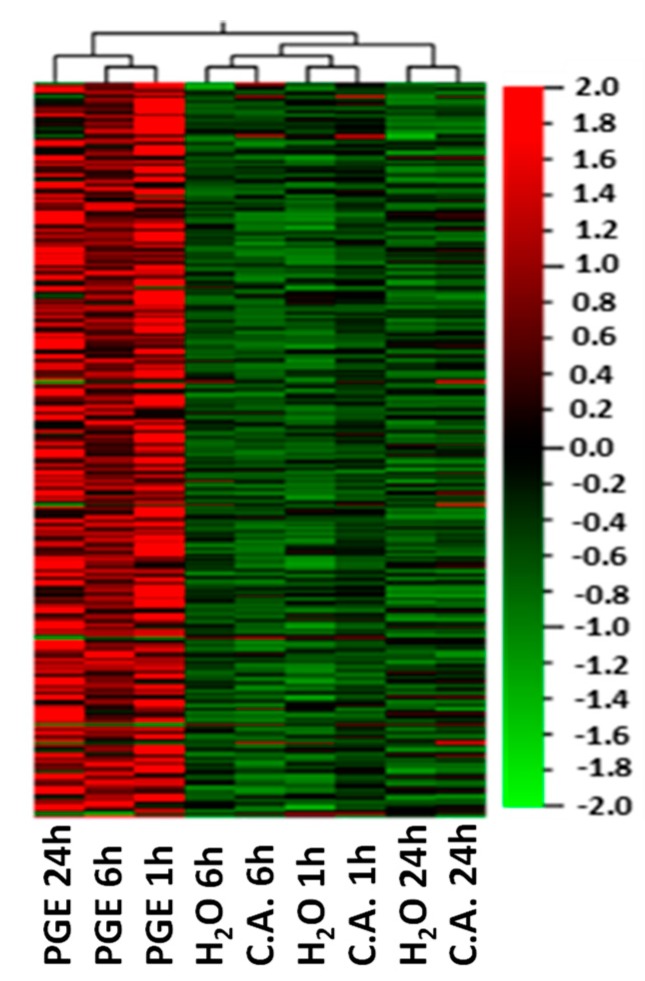
Hierarchical clustering heatmap of differentially expressed genes (DEGs) in orange fruit treated with PGE, citric acid (C.A.) or water (H_2_O) 1, 6 and 24 h post treatment (hpt). Colors indicate the level of expression as indicated in the scale on the right side of the figure.

At all sampling time points (1, 6, and 24 h), the great majority of the DEGs (273) were upregulated in the PGE-treated fruit as compared to water and citric acid, since only a small fraction of genes (8) was down-regulated (Figure 1). Overall, differences between control and PGE-treated fruits increased over the time since the expression level of the upregulated DEGs tended to increase while the downregulated genes showed an opposite expression pattern.

**Figure 2:**
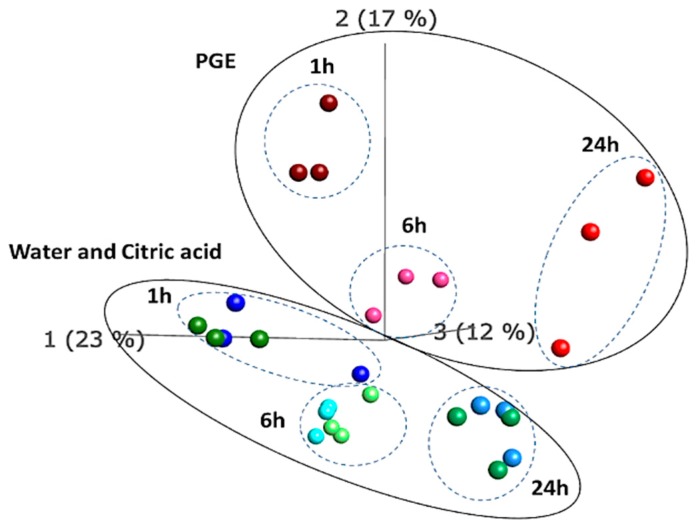
Principal Component Analysis (PCA) of all transcripts from oranges treated with PGE, citric acid or water (control) and analyzed 1, 6 and 24 h post treatment (hpt).

Furthermore, multivariate Principal Component Analysis (PCA) revealed a clustering of the transcriptomes into two groups where the PGE-treated samples were distinctly separated from the control ones (water and citric acid). The clusters representing samples receiving different treatments were further divided into three sub clusters, corresponding to the sampling time i.e., ‘1h’, ‘6h’ and ’24 h’ (Figure 2).

### 3.1. Gene Ontology Enrichment

**Figure 3:**
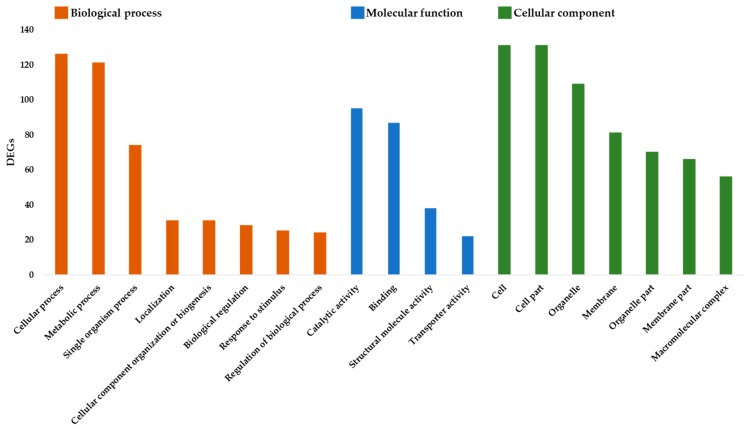
Functional annotation of the differentially expressed genes using Gene Ontology terms.

The GO terms and metabolic pathways of the DEGs were identified by performing functional enrichment analyses. In total, 253 genes were annotated with 1233 GO terms and were assigned to biological process, cellular component, or molecular function (Figure 3). Among the “Biological process” category, the prominent functional groups for both induced and repressed genes were related to the cellular process (126 DEGs), metabolic process (121), single organism process (74), cellular component organization (31), and localization (31). While for the ‘Molecular function’ category, most of the terms belonged to the catalytic activity (95) and binding groups (87). Cell (131), Cell part (131) and Organelle (109) were the most enriched groups in the ‘Cellular component’ category.

### 3.2. KEGG Pathways

KEGG pathway enrichment analysis of the up-regulated and down-regulated genes after PGE treatment showed the involvement of 35 metabolic pathways (Table 2). Among these pathways, 34 were up-regulated while only 1 pathway involved in monoterpenoid biosynthesis was down-regulated. Overall, a large pool of transcripts fell within the area of primary metabolism, i.e., carbohydrate and energy metabolism (16 pathways), amino acid metabolism (4 pathways), and nucleotide metabolism (2 pathways), while other transcripts were mapped to the area of secondary metabolites biosynthesis (6 pathways), and xenobiotics biodegradation and metabolism (6 pathways). Several identified genes translated to enzymes that were involved in multiple pathways. In other cases, multiple enzymes were found to be activated within the same pathways after PGE treatment.

## 4. Discussion

In the present study, a transcriptomic analysis was conducted to evaluate the impact of PGE on the expression of genes in oranges treated at different intervals after treatment (1, 6 and 24 hpt). PGE treatment significantly influenced the gene expression (253 DEGs) compared to the control, while citric acid, commonly utilized to stabilize the extract, did not have any impact. Importantly, a significant impact was revealed at all investigated time points, including 1 hpt, indicating a very quick response of the host tissue. The enrichment analysis showed the involvement of genes mainly in the catalytic and metabolic processes. These results were in accordance with the KEGG analysis where pathways identification revealed that PGE acts entirely on the metabolic pathways of the orange fruit. Among the 35 metabolic pathways, 34 were activated while only 1 pathway, involved in monoterpenoid biosynthesis, was down-regulated. Most of the enriched pathways were involved in primary metabolism, followed by secondary metabolite biosynthesis and xenobiotic metabolism.

The upregulation of primary metabolism indicates an increased demand for energy and biosynthesis, which in turn may modulate signal transduction cascades that lead to plant defense responses [21]. For instance, cysteine and methionine metabolism contained the most up-regulated genes comparing to other pathways, with 6 DEGs coding for 6 different enzymes. Cysteine and methionine are known to be very sensitive amino acids to almost all forms of reactive oxygen, and their metabolism has a crucial role in oxidation resistance in plants [22]. Similarly, 6 DEGs coding for 3 enzymes were up regulated in the oxidative phosphorylation step. This pathway is important for producing cellular energy, which results in the activation of the host defense mechanisms and suppression of the pathogen colonization of the host tissue. Similarly, we found an upregulation of 5 enzymes, involved in the carbon fixation pathways, which are important for the synthesis of new molecules (metabolites) [23]. In addition, among the activated genes, 3 DEGs coded for a very important enzyme, Glutathione S-transferases (GST, ec:2.5.1.18). This enzyme has variety of functions in plant metabolism, but is usually over-expressed after a pathogenic infection [24]. Particularly, it plays major role in plant susceptibility to fungal infection where it is involved in the detoxification step of lipid hydroperoxides produced by peroxidation of membranes. Studies showed its involvement in plant defense signaling, the NPR1-independent SA-mediated pathway [25], hypersensitive reaction and the increase of secondary metabolite production [26]. Other important carbohydrate and energy pathways were also highly upregulated such as ‘Pentose phosphate’, ‘Pyruvate metabolism’, ‘Glycolysis/Gluconeogenesis’, etc. These pathways were reported to be involved in the oxidase activity responsible for production of ROS [23,27]. Therefore, the high transcription level of genes involved in primary metabolism providing energy and intermediate components explains the induction and overexpression of other metabolisms including xenobiotic metabolism and secondary metabolite biosynthesis.

The high upregulation of a battery of genes involved in primary metabolism was accompanied by a high expression of a subset of genes implicated in key pathways of secondary metabolism biosynthesis. The activation of this metabolism reconfirms the assumption of potential involvement of plant defense mechanism triggered by PGE treatment. Particularly, phenylpropanoid biosynthesis, one of the most important components of the plant defense system, was significantly up-regulated after PGE treatment [28]. Phenylpropanoids exhibit a broad spectrum of antimicrobial activity, play a major role as chemical or physical barriers against plant infections, and as signal molecules involved in local and systemic plant defense mechanisms. They participate in the formation of secondary resistance metabolites and are precursors to flavonoids, isoflavonoids, and stilbenes which were also activated following PGE treatment [29]. Many of these metabolites have an antifungal effect, and their overproduction by the plant is considered to be part of a specific antimicrobial defense system [30].

Interestingly, a pathway producing terpenoid volatiles (monoterpenoid biosynthesis pathway) was the only downregulated pathway in this study. Terpenoid volatiles are emitted by plants to communicate with the environment. In sweet orange, these terpenoids are most importantly D-Limonene, a monocyclic monoterpene, which accounts for approximately 97% of the total terpenes in oil glands of orange flavedo [31]. The D-limonene down-regulation was reported to be tightly associated with the activation of the defense responses in the fruit. Rodríguez and co-workers (2014) [32] showed that the downregulation of D-limonene is followed by the up-regulation of genes involved in disease resistance genes.

The analysis also revealed that 9 enzymes, detected in the induced pathways, are also implicated in the biosynthesis of antibiotics. This means that the produced secondary metabolites are explicitly antibiotic substances. Considering that antibiotics are phytochemicals that are known to have antimicrobial and antiviral properties in plants [30,33], the induction of the production of these components might be one of the main mechanisms of action of PGE, especially that its activation continued to increase until 24h post treatment. This induction could be explained mainly by the high concentration in PGE of phenolic compounds [7]. Phenolic components are considered very potent antimicrobial agents that exert a direct effect on the pathogen by the suppression of microbial enzyme systems, increasing the permeability of the cell, etc. [11]. However, phenolics were also reported to induce resistance in the plant. A study on quercetin, a polyphenol that is found in fruits, vegetables, herbs, etc. [34], showed that quercetin application induces resistance in plants and fruits by acting on the transcription level of defense genes [12,13]. Therefore, this suggests that the PGE has a dual mode of action by directly affecting microbial growth, as a phenolic substance, as well as inducing several defense-related gene responses in plants. The co-existence of more than one mechanism of action is considered an important feature to increase efficacy and ensure high levels of protection under different conditions and in different phases of the disease cycle [14]. In particular, the activation of resistance responses may protect commodities from future wound infections, avoid the establishment of latent infections and restrict fungal growth and sporulation. In this context, the results of the present study support previous speculations of the primary role of induced resistance in the persistent efficacy of PGE after its application [8]. Furthermore, its curative effect may be related to the rapid activation of responses that reduce or block the ongoing fungal colonization [10].

The results also showed an overall high expression of defense genes involved in xenobiotic metabolism in oranges treated with PGE. Xenobiotics are foreign chemical contaminants that can be absorbed and accumulated in plant cells [35]. To detoxify these components, the plant induces the expression of several genes involved in the xenobiotic metabolism. The activation of 5 pathways responsible for plant detoxification, and more specifically involving cytochrome P450 and glutathione S-transferases (GSTs), suggests a fruit response to the PGE treatment. In other words, the plant seems to be able to detoxify the extract, and this process should be very effective since PGE did not cause any symptoms of phytotoxicity in treated organs [8,10].

## 5. Conclusions

In conclusion, the results of the present study provide a comprehensive picture of the impact of PGE on the gene expression of treated oranges, highlighting the induction of multiple metabolic responses. These responses are likely to collectively implement a defense system capable of counteracting fungal infections. In particular, GO analysis and pathway mapping of the DEGs showed the induction of important defense pathways, including the phenylpropanoid pathway. However, the massive up-regulation of genes suggest that the induction of defense mechanisms by PGE might be energetically costly for the fruit, which could lead to massive redistribution of energy resources. This would not be an issue in fruits or other mature organs but may be for young growing organs or plantlets. Future investigations will be needed to evaluate these aspects and to experimentally determine the role and function of specific differentially regulated genes in order to dissect their participation in the resistance to pathogens.

## Figures and Tables

**Table 1:** Summary of the results of transcriptomic analysis on oranges treated with PGE, citric acid or water (control) and analyzed 1, 6 and 24 h post treatment (hpt).

Treatment	Replicates	Sampling Time (hpt)	Read 1	Read 2
Citric acid	R1	1	16509545	16509545
	R2	1	10819465	10819465
	R3	1	12792832	12792832
	R1	6	11027909	11027909
	R2	6	14228323	14228323
	R3	6	8719836	8719836
	R1	24	15550388	15550388
	R2	24	24863143	24863143
	R3	24	11287417	11287417
H_2_O	R1	1	13733858	13733858
	R2	1	16898374	16898374
	R3	1	9761332	9761332
	R1	6	22501980	22501980
	R2	6	20726432	20726432
	R3	6	20954767	20954767
	R1	24	15517302	15517302
	R2	24	18455542	18455542
	R3	24	20239137	20239137
PGE	R1	1	9514582	9514582
	R2	1	8451256	8451256
	R3	1	12624217	12624217
	R1	6	11596104	11596104
	R2	6	14399695	14399695
	R3	6	10650424	10650424
	R1	24	13176031	13176031
	R2	24	9556111	9556111
	R3	24	9187532	9187532

**Table 2:** List of KEGG pathways of orange fruits treated with PGE and their corresponding genes.

Category	Pathway	Number of Genes	Enzymes in Pathway
Carbohydrate and Energy metabolism	Glycolysis/Gluconeogenesis	3	ec:5.3.1.1, ec:4.1.2.13, ec:2.7.2.3
	Pyruvate metabolism	3	ec:1.1.1.37, ec:4.4.1.5, ec:3.1.2.6
	Pentose phosphate pathway	3	ec:2.7.1.15, ec:2.2.1.2, ec:4.1.2.13
	Glyoxylate and dicarboxylate metabolism	2	ec:1.1.1.37, ec:1.1.3.15
	Fructose and mannose metabolism	2	ec:5.3.1.1, ec:4.1.2.13
	Pentose and glucuronate interconversions	2	ec:1.1.1.22, ec:4.2.2.2
	Amino sugar and nucleotide sugar metabolism	1	ec:1.1.1.22
	Inositol phosphate metabolism	1	ec:5.3.1.1
	Ascorbate and aldarate metabolism	1	ec:1.1.1.22
	Citrate cycle (TCA cycle)	1	ec:1.1.1.37
	Oxidative phosphorylation	6	ec:1.10.2.2, ec:1.9.3.1, ec:1.6.5.3
	Carbon fixation pathways in prokaryotes	1	ec:1.1.1.37
	Carbon fixation in photosynthetic organisms	4	ec:1.1.1.37, ec:5.3.1.1, ec:4.1.2.13, ec:2.7.2.3
	Methane metabolism	2	ec:1.1.1.37, ec:4.1.2.13
	Nitrogen metabolism	1	ec:1.7.1.1
	Sulfur metabolism	5	ec:3.6.2.1, ec:2.5.1.48, ec:2.7.7.4, ec:2.7.1.25
Lipid metabolism	Glycerolipid metabolism	1	ec:3.1.1.3
Nucleotide metabolism	Purine metabolism	5	ec:3.6.1.3, ec:2.7.7.4, ec:2.7.4.6, ec:2.4.2.7, ec:2.7.1.25
	Pyrimidine metabolism	1	ec:2.7.4.6
Amino acid metabolism	Cysteine and methionine metabolism	6	ec:1.1.1.37, ec:2.5.1.6, ec:2.1.1.14, ec:1.13.11.54, ec:3.3.1.1, ec:2.5.1.48
	Phenylalanine metabolism	1	ec:2.1.1.104
	Selenocompound metabolism	4	ec:2.1.1.14, ec:2.5.1.48, ec:2.7.7.4
	Glutathione metabolism	4	ec:2.5.1.18, ec:1.11.1.15
Biosynthesis of secondary metabolites	Antibiotic biosynthesis	10	ec:1.1.1.37, ec:1.1.3.15 ec:2.5.1.48, ec:2.2.1.2, ec:5.3.1.1, ec:2.7.7.4, ec:4.1.2.13, ec:2.7.4.6, ec:2.7.2.3
	Monoterpenoid biosynthesis	2	ec:4.2.3.20
	Phenylpropanoid biosynthesis	4	ec:1.11.1.7, ec:2.1.1.104
	Flavonoid biosynthesis	1	ec:2.1.1.104
	Monobactam biosynthesis	2	ec:2.7.7.4
	Stilbenoid, diarylheptanoid and gingerol biosynthesis	1	ec:2.1.1.104
Xenobiotics biodegradation and metabolism	Fluorobenzoate degradation	1	ec:3.1.1.45
	Toluene degradation	1	ec:3.1.1.45
	Metabolism of xenobiotics by cytochrome P450	3	ec:2.5.1.18
	Drug metabolism - cytochrome P450	3	ec:2.5.1.18
	Drug metabolism - other enzymes	2	ec:3.1.1.1
	Chlorocyclohexane and chlorobenzene degradation	1	ec:3.1.1.45
